# Sex-Based Risk Evaluation in Acute Coronary Events—A Study Conducted on an Eastern-European Population

**DOI:** 10.3390/medicina61071227

**Published:** 2025-07-06

**Authors:** Svetlana Mosteoru, Nilima Rajpal Kundnani, Abhinav Sharma, Roxana Pleava, Laura Gaita, Dan Ion Gaiță

**Affiliations:** 1 Ph.D. School Department, “Victor Babes” University of Medicine and Pharmacy, 300041 Timisoara, Romaniasharma.abhinav@umft.ro (A.S.); 2 Research Centre of Timisoara Institute of Cardiovascular Diseases, “Victor Babes” University of Medicine and Pharmacy, 300041 Timisoara, Romania; knilima@umft.ro (N.R.K.);; 3 University Clinic of Internal Medicine and Ambulatory Care, Prevention and Cardiovascular Recovery, Department VI—Cardiology, “Victor Babes” University of Medicine and Pharmacy, 300041 Timisoara, Romania; 4 Närhälsan Solgärde Health Care Center, 44240 Kungälv, Sweden; 5 Department of Internal Medicine II, “Victor Babes” University of Medicine and Pharmacy, Eftimie Murgu Sq. No. 2, 300041 Timisoara, Romania; 6 “Pius Brînzeu” Emergency County Hospital, 300723 Timisoara, Romania

**Keywords:** cardiovascular risk factors, acute coronary syndrome, women, sex inequality

## Abstract

*Background and Objectives:* Cardiovascular (CV) diseases account for about 32% of deaths in women, with differing risk factors between women and men. Our study aimed to compare sex-related risk factors and comorbidities in patients at very high CV risk. *Materials and Methods:* We consecutively enrolled adult patients hospitalized for myocardial infarction or unstable angina at a tertiary referral center in western Romania between October 2016 and June 2017. A total of 299 adults underwent clinical and biochemical evaluations between 6 months and 2 years after their coronary event. We assessed patients’ specific characteristics, comorbidities, and risk factors. *Results*: Women made up only a quarter of the survey participants (74 women, 24.7%) and were generally older (63.32 ± 9.3 vs. 60.51 ± 9.3, *p* = 0.02) and more obese (31.20 ± 6.0 vs. 29.48 ± 4.9, *p* = 0.02). There were no significant differences in the prevalence of hypertension, diabetes, dyslipidemia, chronic kidney disease, or peripheral artery disease, though women had slightly higher rates for most comorbidities. Regarding smoking habits, both groups had high percentages of current and former smokers, with women being significantly less likely to smoke (20.9% vs. 44.6%, *p* = 0.003). Multivariable logistic regression adjusting for age, BMI, smoking status, diabetes, and eGFR revealed that sex was not a statistically significant independent predictor for myocardial infarction, PCI, or CABG. *Conclusions*: We observed that women with previous coronary events had a worse risk factor profile, while there were no significant sex differences in overall comorbidities. Risk factor control should be based on sex-specific prediction models.

## 1. Introduction

Cardiovascular diseases represent the most common cause of mortality and morbidity in Europe, accounting for 45% of all deaths [1], while coronary artery disease (CAD), the most common form of CVD, represents 20% of all mortality in Europe [2]. It was believed that CAD affects males more frequently. Females have a similar rate of prevalence only after the age of 65 due to the so-called hormonal protection effects. However, recent data have come to challenge this view, as more information is coming to light, showing that both sexes are similarly affected by this disease. According to the latest data, CAD represents 16% of all premature mortality under the age of 75 for females and 18% among men, while mortality rates caused by CAD under the age of 65 are 16% among men and 10% among women [2]. However, there are large differences between European countries, some having almost 10 times greater rates than others in both men and women [3]. Such is the case of Romania, where the standardized death rates due to ischemic heart diseases are 347.6/100,000 inhabitants for males and 231.8/100,000 inhabitants for females [3].

Romania, once considered to be a large East European country, has been gradually declining in regard to population, from 20,147,657 inhabitants in the 2011 census, to 19,261,714 inhabitants in 2020, and it is predicted to reach 15,502,837 by 2050. Additionally, it is predicted that the female population is expected to decrease by 21.0% while the male population by 18.2% by 2050 [4].

Although healthcare professionals constantly emphasize awareness of CAD, females are less likely to recognize symptoms of the disease, causing a delay in contacting their physicians due to the absence of angina symptoms, or a less specific chest pain pattern and more nausea, dyspnea, indigestion and jaw, head and back pain, thus delaying the treatment [5]. Moreover, the ability to observe cardiovascular risk factors are considered to be influenced by age and educational level [6]. A deeper understanding of sex differences in patient awareness is necessary to optimize CHD risk factor control in daily clinical practice.

The aim of our study was to compare sex-based risk factors and comorbidities in a high-risk CV group of participants from a middle-income country, based on data acquired from the EUROASPIRE V survey, from the Romanian database.

The EUROASPIRE V (European Survey of Cardiovascular Disease Prevention and Diabetes) survey represents one of the five cross-sectional surveys developed by the European Society of Cardiology under the EORP (EUR Observational Research Program) between 2016 and 2017, the aim of which was to evaluate the implementation of cardiovascular disease prevention guidelines in clinical practice. Twenty-seven countries participated in this survey, including Romania.

## 2. Materials and Methods

We conducted a cross-sectional descriptive study in which our main objective was to assess the sex-based differences in patients hospitalized due to an acute coronary event.

### 2.1. Patient Recruitment

Patients were retrospectively identified through hospital discharge lists and then called for an interview. Patients between 18 and 80 years old with a recent history of hospitalization for an acute coronary syndrome (myocardial infarction (MI)) or unstable angina (UA)), were enrolled in our study from a tertiary referral center from western Romania. All the patients had undergone either elective or emergency percutaneous coronary intervention (PCI), or coronary artery bypass grafting (CABG) in the past 6–24 months, prior to study enrolment. Eligible patients were invited to participate in the study, and the visits took place from October 2016 until June 2017. The eligibility of the patients was determined based on the 2016 Guidelines on CVD prevention and the 2017 Guidelines on Management of Acute Myocardial Infarction in Patients presenting with ST segment elevation. The study received the local ethics committee approval, from the Ethics Committee of the Institute for Cardiovascular Diseases Timisoara, (6978/30 September 2016) and the principles of the Helsinki Declaration were respected. All participants signed written informed consent which was stored in the patient file.

### 2.2. Data Collection

Patients satisfying inclusion/exclusion criteria were interviewed at the study visit by trained research staff that collected demographic and anthropometric data, measured blood pressure and breath carbon monoxide (CO) and administered the questionnaires. Demographic data included age, sex, smoking status and anthropometric measures included height, weight, abdominal and neck circumference. The data collection also included information on the type of procedure performed (PCI or CABG), the primary diagnosis (MI or UA), as well as the left ventricular ejection fraction (LVEF) measured by echocardiography at discharge. The medical history was carefully assessed for all the patients with an emphasis on the presence of several specific comorbidities (type 2 diabetes, arterial hypertension, dyslipidemia) based on patients’ self-reports and review of concomitant medication and available medical reports (e.g., discharge letters).

Systolic and diastolic blood pressures (BP), as well as heart rate readings, were obtained using an automatic digital sphygmomanometer (Omron M6). The BP was measured in both arms to detect possible significant differences, with the cuff positioned at the level of the heart. If a significant difference was not detected, the arm with the highest value was used for reference (according to the European Society of Cardiology Guidelines) [7]. To chemically validate self-reports of smoking status, we analyzed breath CO using a Bedfont Smokerlyzer device(Bedfont® Scientific Ltd, Station Road, Harrietsham, Maidstone, Kent, ME17 1JA, England). Fasting venous blood samples were drawn on the morning of the study visit and were used to evaluate the level of plasma glucose and serum creatinine, and patients provided a morning urine sample for the evaluation of urinary albumin/creatinine ratio. The data collected were reviewed by physicians, and the estimated glomerular filtration rate (eGFR) was calculated using the CKD-EPI formula [8]. The sample was divided into two groups based on sex, subsequently named the male and female groups.

### 2.3. Statistical Analysis

Data were stored and managed using Microsoft Excel 2010, and statistical analyses were performed using SciStat v2013. Mean and standard deviations expressed to two decimal places were used for scale-type data, and group percentage was used for ordinal data. The Student *t*-test and Mann–Whitney test were used to compare parametric and non-parametric data, respectively, and the statistical significance was set at α = 0.05.

Additionally, multivariable logistic regression models were constructed to assess the independent effect of sex on the outcomes of interest (MI, PCI, CABG), adjusting for clinically relevant covariates: age, BMI, smoking status, diabetes mellitus, and estimated glomerular filtration rate (eGFR). Odds ratios (ORs), 95% confidence intervals (CI), and *p*-values were reported. Statistical significance was defined as *p* < 0.05.

Furthermore, we focused on the admission diagnoses (myocardial infarction [MI] vs. unstable angina [UA]) and procedures (CABG vs. PCI) between males and females to provide odds ratios (OR) for these categorical variables.

## 3. Results

### 3.1. Patient Characteristics

**Figure 1:**
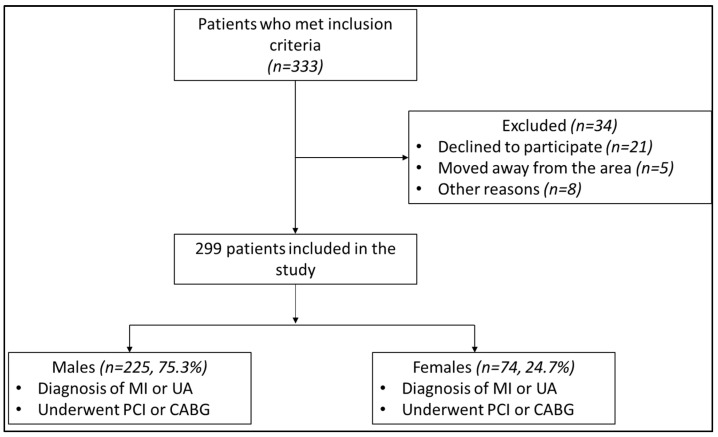
Flowchart of the study design MI—myocardial infarction; UA—unstable angina; PCI—percutaneous coronary intervention; CABG—coronary artery by-pass grafting.

A total of 333 patients met the inclusion criteria and were invited to participate in the study between October 2016 and June 2017; 21 patients declined to participate, 5 could not attend as they moved away from the area and 8 died. The remaining 299 patients (225, 75.3% male) with a recent history of myocardial infarction (48.8%, 146 patients) or unstable angina (51.2%, 153 patients) were enrolled in the study (Figure 1). The mean age of the study group was 61.21 ± 9.4 years, the majority being overweight with a mean body-mass index (BMI) of 29.9 kg/m^2^ (Table 1). Women accounted for only a quarter of the survey participants (74 women, 24.7%) and were generally older (60.51 ± 9.3 men vs. 63.32 ± 9.3 women, *p* = 0.026) and more obese (29.48 ± 4.9 men vs. 31.20 ± 6.1 women, *p* = 0.029) (Table 2 and Table 3).

Categorical Variables (Admission diagnosis and procedures)

**1.**  **Myocardial Infarction (MI) vs. Unstable Angina (UA) by sex**

**Table 2:** Myocardial Infarction vs. Unstable Angina by sex.

	Male	Female
MI	116	30
UA	109	44

- Odds Ratio (OR): (116/109) (30/44) = 116 × 44/109 × 30 ≈ 1.56
- 95% Confidence Interval (CI): 0.92 − 2.650.92 − 2.65
- Interpretation: Males had 1.56 times higher odds of MI compared to UA than females, but this association was not statistically significant (*p* = 0.100).

**2.**  **CABG vs. PCI by sex**

**Table 3:** CABG vs. PCI by sex.

	Male	Female
CABG	53	11
PCI	172	63

- **Odds Ratio (OR):** (53/172) (11/63) = 53 × 63/172 × 11 ≈ 1.77
- **95% Confidence Interval (CI):** 0.87 − 3.59.
- **Interpretation:** Males had 1.77 times higher odds of undergoing CABG compared to PCI than females, but this association was not statistically significant (*p* = 0.114).

There was a statistically significant difference between mean height and weight between the two groups, males and females, as well as a significant difference regarding neck circumference. There was no difference between the mean systolic blood pressure, while there was a significant difference noted regarding mean diastolic blood pressure. There was also no statistically significant difference between the two groups regarding heart rate, mean carbon monoxide levels, mean glucose levels or ejection fraction. However, when analyzing the kidney function, we observed a statistically significant difference between males and females regarding mean serum creatinine levels and albumin/creatinine ratio, higher among men.

A total of 51% of the patients had been admitted to hospital due to unstable angina and 49% due to myocardial infarction. The vast majority of patients (79%) were treated by PCI.

### 3.2. Age-Adjusted Sex Comparisons

To further explore whether observed sex differences were independent of age, we performed logistic regression analyses adjusting for age (Table 4). The age-adjusted odds ratios (OR) showed that females had 31% lower odds of having had a myocardial infarction (OR = 0.69; 95% CI: 0.41–1.19; *p* = 0.18) compared to males, although the difference was not statistically significant. Regarding procedures, females had 83% higher odds of undergoing PCI rather than CABG (OR = 1.83; 95% CI: 0.90–3.74; *p* = 0.097), while they had 44% lower odds of undergoing bypass surgery compared to males (OR = 0.56; 95% CI: 0.27–1.14; *p* = 0.108), but again without reaching statistical significance.

These results indicate that although some sex differences were observed in crude comparisons, after adjusting for age, the associations were attenuated and did not reach statistical significance.

### 3.3. Smoking Habits

While there was no statistically significant difference regarding active smoking status or years spent smoking between men and women, there is a significant difference for non-smokers (in favor of women) (20.9% women vs. 44.6% men, *p* = 0.003) and former smokers (in favor of men) (40.9% men vs. 23.0% women, *p* = 0.049) (Table 5).

### 3.4. Comorbidities

**Figure 2:**
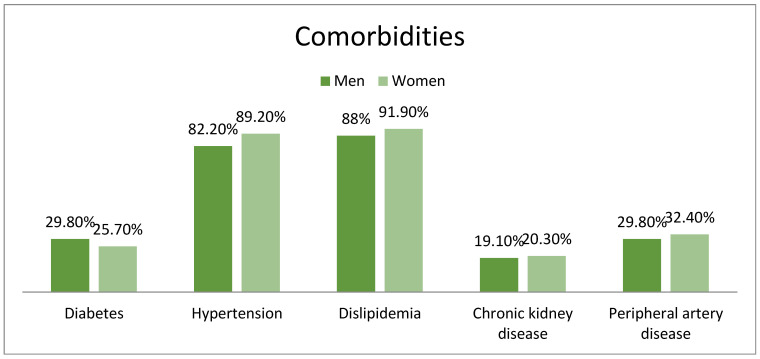
Comorbidities.

There were no significant differences in the prevalence of hypertension (82.2% men vs. 89.2% women, *p* = 0.15), diabetes mellitus (29.8% men vs. 25.7% women, *p* = 0.49), dyslipidemia (88% men vs. 91.9% women, *p* = 0.35), chronic kidney disease (19.1% men vs. 20.3% women, *p* = 0.82) or peripheral artery disease (29.8% men vs. 32.4% women, *p* = 0.66), although women had a higher percentage for most comorbidities (Figure 2).

### 3.5. Multivariate Logostic Regression

To further assess sex as an independent predictor, we performed multivariable logistic regression adjusting for age, BMI, smoking, diabetes, and eGFR.

For myocardial infarction, smoking was the only significant predictor (OR = 2.62, 95% CI: 1.32–5.19, *p* = 0.006). Sex was not significant (OR = 0.69, 95% CI: 0.34–1.45, *p* = 0.336). For PCI and CABG, none of the covariates were statistically significant predictors, including sex (*p* > 0.2 for all). These findings reinforce that while crude sex differences exist, they are attenuated after adjusting for major risk factors.

**Figure 3:**
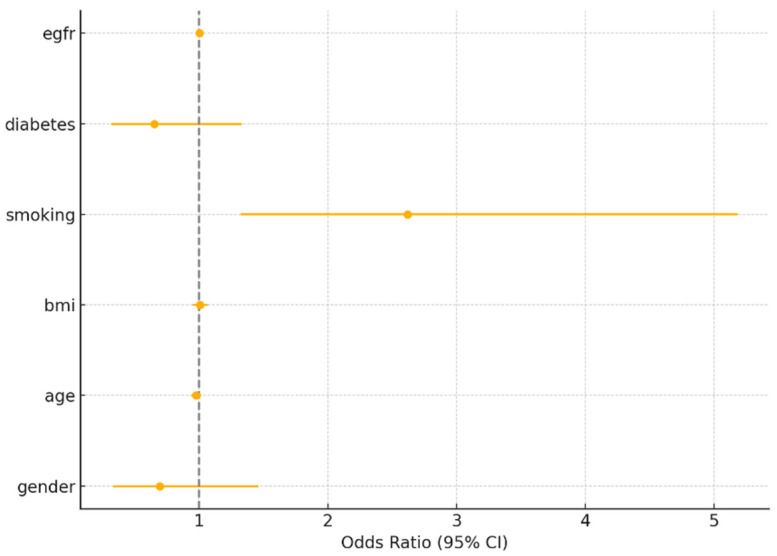
Odds ratios for myocardial infarction (MI).

**Figure 4:**
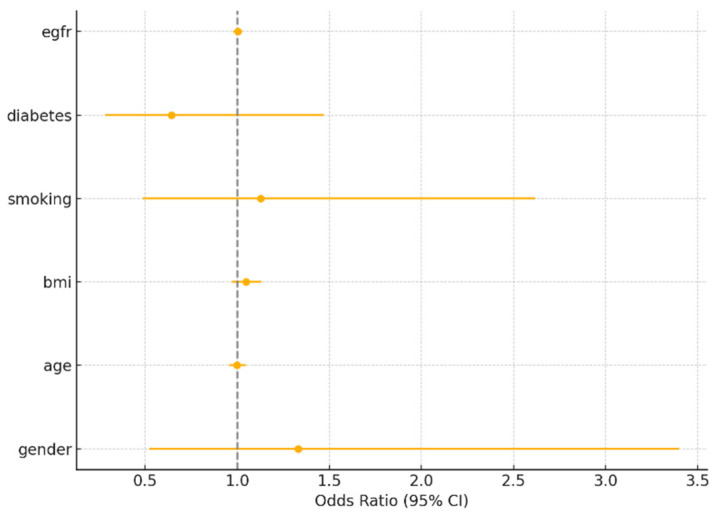
Odds ratios for percutaneous coronary intervention (PCI).

**Figure 5:**
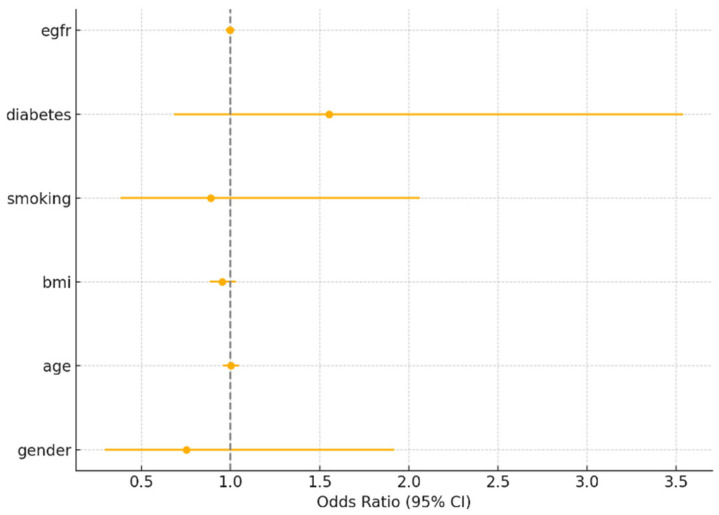
Odds ratios for coronary artery bypass grafting (CABG).

Below are the tables with the results (Table 6, Table 7 and Table 8) (Figure 3, Figure 4 and Figure 5).

### 3.6. Follow-Up

All patients were followed up for one year and the survival rate was 100%, without any additional major cardiovascular events during this period.

## 4. Discussion

The current study aims to provide insight into sex-based differences regarding risk factor control in a population from a middle-income Eastern European country who has experienced an acute coronary event. Patients were enrolled and analyzed by European Prevention Guidelines currently in practice at the time of enrollment, as per EUROASPIRE survey standards. Although previous studies have shown that women are more likely to have a poorer risk factor profile than men [9], our results suggest that both sexes are similarly affected by diabetes mellitus, arterial hypertension, dyslipidemia, chronic kidney disease or peripheral artery disease.

To better understand whether sex differences were attributable to age disparities, we performed age-adjusted analyses. Logistic regression models adjusting for age demonstrated that women had lower odds of presenting with myocardial infarction and bypass surgery compared to men, and higher odds of undergoing PCI, although none of these differences reached statistical significance. This suggests that some of the sex disparities initially observed could be partly explained by the older average age of women in the cohort. These findings emphasize the importance of adjusting for age when analyzing sex-based differences, especially in populations where women typically present at older ages with cardiovascular disease.

Moreover, our extended multivariable analysis confirmed that smoking remains a strong predictor of myocardial infarction, while sex was not independently associated with MI, PCI, or CABG after adjusting for key covariates. These results are consistent with prior studies and suggest that observed sex disparities may be mediated by differential risk factor profiles, especially smoking behavior. Thus, prevention efforts should target modifiable risks like smoking more than sex alone.

Analyses have been made on the EUROASPIRE cohort showing only subtle differences in the medical treatment of coronary heart disease patients irrespective of sex, however, with major differences in risk factors occurrences and target attainments in women for EUROASPIRE III [10]. EUROASPIRE IV showed that the clustering of risk factors was higher in females, with the gap decreasing in younger age or higher education [11], while data from EUROASPIRE V showed that there are few sex differences in patient risk factor awareness, in disfavor of women [12]. The significance of thoroughly understanding risk factors has been well documented, as it is crucial for optimizing risk factor management in CHD patients and reducing the likelihood of recurrent coronary events. However, previous studies have shown that many CHD patients remain unaware of their risk factors [12].

Our results showed that women were generally older and more obese than men, although they smoked less, similar to data found by Vynckier et al. [12]. Moreover, these sex differences were more pronounced in middle-income countries as compared to high-income countries, where women were less likely to be non-obese than men [13]. However, a sex difference regarding smoking was not noticeable in high-income countries compared to middle-income countries, possibly because of the smoking culture, where an increasing number of younger females have started smoking in high-income countries [13].

Women have a 25% increased risk for CAD due to smoking, as compared to men [13]. More so, the combination of oral contraceptives with smoking in women of fertile age increases the risk for acute myocardial infarction, stroke, or venous thromboembolism [14]. Women tend to have poorer health status than men at the onset of acute coronary syndrome. Smoking and diabetes appear to be factors that are more significant for women than for men, with the cardiovascular risk associated with smoking being particularly high in young and middle-aged women. Differences in socioeconomic status, psychosocial stress, and mental health may further contribute to cardiovascular risk in women. Research suggests that depression and perceived stress are strong predictors of cardiovascular risk, especially in younger and middle-aged women [15].

The impact of obesity in the development of CAD is more significant in women than in men, according to results from the Framingham Heart Study, where obesity increased the relative risk for CAD by 64% in women as compared to 46% in men [16].

Although our results showed that known chronic kidney disease as a comorbidity among men and women did not appear as a significant sex difference, the difference in mean serum creatinine levels and albumin/creatinine ratio was shown to be statistically significant between the two sexes. The Global Burden of Disease, Injuries and Risk Factors Study (GBD) has previously shown considerable sex disparities in the prevalence of CKD throughout the world in disfavor of women. These differences were observed not only in CKD stages with decreased glomerular filtration rate (GFR), but also in the presence of albuminuria with normal GFR [17].

Elevated albumin/creatinine ratio levels are associated with an increased risk of cardiovascular events in both men and women. However, the nature of these associations can differ by sex. In women, a higher albumin/creatinine ratio is a significant predictor of all-cause and cardiovascular mortality. In contrast, in men, an elevated albumin/creatine ratio is more strongly associated with hospitalization for heart failure and myocardial infarction. These findings suggest that while an elevated albumin/creatinine ratio is a critical risk factor for cardiovascular events in both sexes, the specific outcomes and their severity may vary between men and women [18].

Since all patients included in the study presented with acute coronary syndrome, it is interesting to note that the vast majority of patients were treated by PCI (79%), and only 64 patients (21%) underwent CAGB. Out of these patients, only 18% were females. Although availability of CABG was equally similar for both men and women, the fact that fewer females underwent this procedure could also be explained by patient choice. Other studies comparing sex-specific differences in patients undergoing PCI have shown that women typically present with less complex coronary artery lesions despite the fact that they are usually older and have many more comorbidities, thus being more likely to undergo PCI rather than CABG [19].

A similar study was recently published involving the comparison of the sex differences in the Belgian population, which is considered to be a high-income country, and also a part of the EUROASPIRE survey, which demonstrated little to no sex differences in the management of CHD patients, although women still presented with a worse risk factor profile, both in Belgium and in other European high-income countries [20].

A study published by Zhao et al. in 2017 regarding sex differences in risk factor management of coronary heart disease across different regions (which included patients from Romania as well) showed that women generally have a poorer risk factor profile and are less likely to achieve therapeutic targets compared to men [21]. However, these results vary depending on region and age. Europe was examined as a region, but no country analysis was performed, giving our study the benefit of being one of the few to assess the situation in an Eastern European population, which may vary compared to Western European populations.

Another study, recently published by Marzà-Florensa et al. regarding results from the SURF II study, showed that patients with higher educational levels were generally more likely to achieve risk factor targets, although these associations varied by country income group and specific risk factor. A higher educational level was linked to meeting smoking targets across all country-income groups. However, in upper-middle-income countries (UMICs), a higher educational level was negatively associated with achieving waist circumference and LDL targets, while in lower-middle-income countries (LMICs), it was negatively associated with meeting BMI targets [22].

The differences in risk factor target attainment by educational level observed in that study could be partly due to variations in risk factor awareness and health literacy. Patients with higher educational levels are more likely to be aware of their risk factors, understand their measured levels and targets, and possess greater health literacy [22].

The EUROASPIRE surveys have extensively compared cardiovascular health metrics across European populations, often highlighting disparities between Eastern and Western Europe. While these comparisons provide valuable insights, a deeper examination of the unique aspects of Eastern European healthcare systems and cultural factors is essential to fully understand the sex disparities observed in cardiovascular health. Cultural norms in Eastern Europe have traditionally emphasized patriarchal values, influencing sex roles and expectations. Additionally, socioeconomic disparities, such as lower income levels and reduced access to education and employment opportunities for women, further exacerbate health inequalities. These factors collectively contribute to a “worse risk factor profile” in women, despite lower reported rates of smoking compared to men [23].

The American Heart Association first launched the “Go Red for Women” campaign in 2004, an event meant to raise awareness about CVD affecting women in general, which was later endorsed by the World Heart Federation and promoted worldwide as an annual event [24]. Despite such endeavors, we have yet to see a consistent improvement in women’s cardiovascular health as shown by this study as well as others. Many more awareness campaigns, as well as perhaps policy adjustments, are needed to change the current status.

### Limitations of the Study

One of the limitations of our study is the small number of females included (24.7%) as compared to males, which may influence the results of the study. However, this has become a challenge for many studies involving sex related differences, as men tend to be the majority in study populations. One of the possible explanations is that more males agree to participate in studies compared to females (out of the 21 patients that declined to participate, 19 were females), as well as the fact that more male patients were admitted to the hospital for acute coronary syndromes. Although the entire population (299 patients) may appear small in size and thus impact the generalizability of the results, it was considered statistically sufficient for the size of our country for the EUROASPIRE survey and no other survey of this magnitude has been conducted so far.

Another limitation of the study could be the fact that the data analyzed were gathered pre-COVID pandemic, which may have changed the landscape of cardiovascular diseases. It is interesting to note that the statistics regarding the incidence of ischemic heart disease between 2011 and 2020 in Romania showed a decrease from 822.1/100,000 inhabitants to 784.3/100,000 inhabitants [4].

A major strength of the study is that it is one of the few involving the Romanian population to investigate sex-based differences in cardiovascular risk factors.

## 5. Conclusions

Although CVD had been traditionally branded a “man’s disease”, females have shown a worse risk factor profile (older age, more obese, higher CO levels, more dyslipidemic) and these differences have begun to be more thoroughly investigated over recent years, as studies regarding sex difference have started to emerge. While our results showed no statistically significant sex differences regarding the overall comorbidities, although women had a higher percentage for most comorbidities, risk factor control needs to follow sex-specific prediction models, and more women should be included in CVD studies. In addition, more studies regarding sex differences are needed in order to thoroughly examine these variations. However, the insights from this study are valuable for helping healthcare professionals adopt a more targeted strategy in the secondary prevention of coronary heart disease.

## Figures and Tables

**Table 1:** Demographic and clinical characteristics.

Variable	All Patients (n = 299)	Males (n = 225)	Females (n = 74)	*p*-Value
Demographics and clinical				
Age, years	61.21 ± 9.4	60.51 ± 9.3	63.32 ± 9.3	**0.026**
Height, m	1.7 ± 0.08	1.73 ± 0.1	1.61 ± 0.1	**<0.001**
Weight, kg	86.59 ± 16.2	88.35 ± 15.7	81.24 ± 16.7	**0.001**
BMI, kg/m^2^	29.9 ± 5.3	29.48 ± 4.99	31.20 ± 6.1	**0.029**
Abdominal circumference, cm	105.19 ± 13.6	105.92 ± 13.5	102.98 ± 13.5	0.107
Neck circumference, cm	38.27 ± 4.8	39.09 ± 4.7/8	35.75 ± 3.8	**<0.001**
Systolic blood pressure, mmHg	136.7 ± 19.8	137.87 ± 20.2	132.98 ± 18.1	0.053
Diastolic blood pressure, mmHg	81.31 ± 11.2	82.13 ± 11.4	79.10 ± 10.3	**0.034**
Heart rate, bpm	70.18 ± 39.8	70.83 ± 45.6	68.21 ± 8.95	0.416
CO, p.p.m. (mean, IQR)	3.0 (2.0)	4.5 (5.7)	4.67 (6.5)	0.908 *
Blood glucose, mmol/L (mean, IQR)	7.0 (2.2)	7.75 (2.71)	7.30 (1.9)	0.125 *
Albumin/creatinine ratio, mg/g (mean, IQR)	6.3 (7.3)	24.19 (79.9)	14.24 (21.5)	**0.042** *
Ejection fraction, % (mean, IQR)	50.0 (5.0)	47.07 (8.1)	48.92 (5.6)	0.121 *
Serum creatinine, mg/dL (mean, IQR)	1.20 (0.3)	1.29 (0.5)	1.10 (0.3)	**<0.001** *
Admission diagnosis and procedures				
Myocardial infarction, n (%)	146 (49)	116 (79.45)	30 (20.54)	0.100
Unstable angina, n (%)	153 (51)	109 (71.24)	44 (28.75)	
CABG, n (%)	64 (21)	53 (82.81)	11 (18)	0.114
PCI, n (%)	235 (79)	172 (73.19)	63 (26.80)	

Note: eGFR—estimated glomerular filtration rate; BMI—body mass index; MI—acute myocardial infarction; UA—unstable angina; PCI—percutaneous coronary intervention; CABG—coronary artery bypass grafting; CO—carbon monoxide; * Continuous variable are expressed as mean ± SD; * Mann-Whitney test * *p*-values in bold are statistically significant.

**Table 4:** Age-adjusted odds ratios (ORs) comparing male vs. female for myocardial infarction, PCI and bypass.

Comparison	Age-Adjusted OR	95% CI	*p* Value	Interpretation
Female vs. male—myocardial infarction	0.69	0.41–1.19	0.18	Women had 31% lower odds of MI than men after adjusting for age, but not significant.
Female vs. male—PCI	1.83	0.90–3.74	0.097	Women had 83% higher odds of undergoing PCI compared to men, but not significant.
Female vs. male—bypass	0.56	0.27–1.14	0.108	Women had 44% lower odds of undergoing bypass compared to men, after age adjustment, not significant.

**Table 5:** Smoking characteristics as based on sex.

	Males (n = 225)	Females (n = 74)	*p* Value
Active smokers, n (%)	86 (38.2)	24 (32.4)	0.538
Former smoker, n (%)	92 (40.9)	17 (23.0)	0.049 **
Non-smokers, n (%)	47 (20.9)	33 (44.6)	0.003 **
Years spent smoking, mean ± SD	32.71 ± 9.1	30.56 ± 10.7	0.387

Note: Continuous variables expressed as mean ± SD; ** *p*-values in bold are statistically significant.

**Table 6:** Myocardial infarction (MI) logistic regression.

Unnamed: 0	Variable	Odds Ratio	95%CI	*p* > [z]
**1**	Gender	0.69	0.33–1.46	0.3363875661672745
**2**	Age	0.98	0.94–1.01	0.2193649896124957
**3**	BMI	1.01	0.95–1.07	0.8361810081296472
**4**	Smoking	2.62	1.32–5.18	0.0056811920613911
**5**	Diabetes	0.65	0.32–1.33	0.2410744833789785
**6**	eGFR	1.0	0.98–1.02	0.8713422945143566

**Table 7:** PCI (percutaneous coronary intervention) logistic regression.

Unnamed: 0.	Variable	Odds Ratio	95%CI	*p* > [z]
**1**	Gender	1.33	0.52–3.40	0.5497505352473568
**2**	Age	1.0	0.95–1.04	0.9498877752218704
**3**	BMI	1.05	0.97–1.13	0.2230201164506067
**4**	Smoking	1.13	0.49–2.62	0.781233636744233
**5**	Diabetes	0.64	0.28–1.47	0.2947966736083327
**6**	eGFR	1.0	0.98–1.03	0.852235818270531

**Table 8:** Coronary artery bypass grafting (CABG) logistic regression.

Unnamed: 0	Variable	Odds Ratio	95%CI	*p* > [z]
**1**	Gender	0.75	0.29–1.92	0.5497505352473575
**2**	Age	1.0	0.96–1.05	0.9498877752218718
**3**	BMI	0.95	0.88–1.03	0.2230201164506084
**4**	Smoking	0.89	0.38–2.06	0.7812336367442339
**5**	Diabetes	1.55	0.68–3.54	0.2947966736083329
**6**	eGFR	1.0	0.97–1.02	0.8522358182705314

## Data Availability

The data will be provided on valid request to the corresponding author.
